# Policy implications of physicians’ attitudes towards being examined by medical students

**DOI:** 10.1186/s13584-025-00711-6

**Published:** 2025-08-13

**Authors:** Ruth Kannai, Tamar Freud, Tsafnat Test, Roni Peleg

**Affiliations:** 1 Siaal Research Center for Family Medicine and Primary care, Department of Family Medicine, The Haim Doron Division of Community Health, Faculty of Health Sciences, Ben-Gurion University of the Negev, POB 653, Beer-Sheva, 8410501 Israel; 2 Clalit Health Services, Jerusalem district, Jerusalem, Israel; 3 Clalit Health Services, Southern District, Beer-Sheva, Israel

**Keywords:** Medical education policy, Family physicians, Physician-patients, Informed consent, Professional boundaries, Clinical teaching assignments.

## Abstract

**Background:**

Physicians who become patients—especially those involved in teaching—hold a dual perspective that may influence their comfort with medical student involvement in their care. Understanding these attitudes is essential for shaping medical education policies related to consent, patient assignment, and professional boundaries. This study explored Israeli family physicians’ willingness to be examined by medical students and examined implications for clinical teaching policy.

**Methods:**

A cross-sectional survey was conducted among Israeli family physicians during June–July 2024. A total of 149 participants completed a structured questionnaire assessing their comfort with students observing, taking medical histories, and performing physical, intimate, and invasive examinations. Data were analyzed using descriptive statistics and chi-square tests to compare subgroups by gender, age, and teaching status.

**Results:**

Among the 149 respondents (mean age 47.2 years; 65.1% female), 92.6% were comfortable with students observing non-intimate exams, but only 29.5% agreed to student presence during intimate exams. Comfort increased slightly if the student was of the same gender (48.3%), but overall acceptance remained low. Female physicians were significantly less willing than males to undergo physical exams by students (63.5% vs. 88.2%; *p* = 0.006) and were more uncomfortable with male students performing intimate exams (48% vs. 28.1%; *p* = 0.014). Physicians aged ≤ 45 were less willing to student observation than those aged ≥ 46 (61.0% vs. 42.3%; *p* = 0.033). Only 16.8% agreed to allow students to perform invasive procedures. Interestingly, teaching physicians were significantly less likely than non-teachers to agree to student presence during intimate exams (29.7% vs. 51.6%; *p* = 0.018).

**Conclusions:**

While student involvement in non-invasive care is widely accepted, substantial discomfort persists with intimate and invasive scenarios—particularly among female and teaching physicians. These findings raise ethical and educational concerns. Institutions should consider: (1) Allowing physician-patients to opt out of student involvement without stigma; (2) Implementing real-time, standardized informed consent for all patients, including physicians; (3) Creating clear guidelines on teacher-student dynamics in clinical care.

**Supplementary Information:**

The online version contains supplementary material available at 10.1186/s13584-025-00711-6.

## Introduction

Clinical teaching takes many forms, including bedside instruction during hospital work rounds, attending rounds, and supervised outpatient settings. In each context, medical students may participate passively—by observing—or actively—by taking histories, performing physical examinations, or carrying out procedures. The degree of involvement varies based on the teaching model, the student’s level of training, and the clinical setting.

Sir William Osler emphasized the value of bedside learning, stating, “Medicine is learned at the bedside and not in the classroom” [1]. The American Medical Association’s (AMA) Code of Medical Ethics also highlights the importance of patient interaction in medical training [2]. However, the obligation to train future physicians must be balanced with patients’ autonomy and their right to choose who is involved in their care.

Numerous studies have examined patients’ attitudes toward medical student participation in clinical care [3–7]. These generally show that patients respond positively to student involvement, particularly when supervision is provided [8–10]. Yet, in many settings, consent for student involvement is assumed rather than explicitly obtained [11, 12].

Physicians who become patients face unique challenges, including role ambiguity, loss of professional identity, and heightened concerns about privacy [13]. Some may experience discomfort that leads them to withhold sensitive information during history taking or physical examinations [14]. These dynamics raise specific concerns about boundaries, especially when physician-patients are familiar with or supervise the students involved.

Understanding how physicians respond to student involvement when they themselves are patients is essential for shaping medical education policy. In teaching hospitals and academic clinics, physicians may be selected—intentionally or unintentionally—as patients for medical student training. Their familiarity with clinical education may lead to stricter expectations regarding consent, a preference for privacy, or discomfort with role reversals. Their attitudes may influence institutional practices regarding student-patient assignments, informed consent procedures, and expectations around professional boundaries.

In Israel, clinical clerkships often involve large student groups rotating through a single department or clinic, increasing the likelihood that patients—including physicians—will encounter students during a visit. This structure heightens the complexity of student-patient interactions and makes physician-patient perspectives particularly relevant to educational policy.

Physicians who become patients raise important policy questions: Should they be excluded from student encounters, even passive observation or routine exams? Should faculty-physicians be treated differently? Should students be allowed to ask intimate questions or perform intimate exams on physician-patients—and does the student’s gender matter? Could some physician-patients, such as those with unique physical findings, be especially valuable for teaching? These considerations underscore the need to explore physician-patient attitudes toward student involvement. This study addresses that gap to inform policy on clinical teaching assignments, consent, and professional boundaries.

## Methods

### Study design and setting

Cross-sectional survey study conducted in Israel during June-July 2024.

### Study population

#### Study population and sampling

This was a convenience sample including.

1. Family physicians from the “Kehiladocs” network (self-managed network of family medicine specialists and residents).
2. Residents and specialists from Clalit Health Services southern district participating in CME sessions.

Both groups completed the questionnaire digitally—one recruited online via email, the other face-to-face but given the same digital link.

### Data collection

Data were collected using an electronic structured research questionnaire created with Qualtrics software. The questionnaire link was distributed via email to relevant groups within the study population or distributed during CME sessions for completion.

### Study tool

We utilized a structured questionnaire designed by researchers based on literature review (Supplementary material 1). The questionnaire was not validated against existing instruments or tested for correlation with actual behavior. Questions used 5-point Likert scales (1–5) assessing willingness to:

- Allow medical student observation during medical sessions (4 questions).
- Permit student anamnesis (medical history)-taking (5 questions).
- Allow student physical examination (4 questions).
- Permit student procedures under supervision (5 questions).

“Intimate examinations” were defined as examinations of genitals, breasts, or rectum. “Minimally invasive procedures” included venipuncture, suturing simple lacerations, or IV insertion.

Additionally, we gathered socio-demographic information from respondents.

### Sample size

The sample size was calculated based on an expected physician willingness rate of 80% ± 5%, informed by the researchers’ prior experience, as no previous studies were found to support this assumption. Assuming a total population of 520 physicians, a 95% confidence level, and a design effect (DEFF) of 1, the required sample size was determined to be 147 using OpenEpi (Version 3.0). This calculation corresponds to a 5% significance level and provides sufficient precision for estimating the proportion of willingness.

### Statistical analysis

We analyzed data using IBS-SPSS software version 29. Categorical variables are presented as frequencies, while continuous variables are presented as averages and standard deviations.

We analyzed physicians’ willingness to involve medical students in their care during training in two ways:

- Categorical level of agreement: Responses to each item on the 1–5 Likert scale were grouped as follows: Disagree (1–2), Neutral (3), Agree (4–5).
- Agreement total score (attitude score): A total score was calculated based on the respondents’ level of agreement across all questionnaire items, as well as for each subcategory: Students passively observing, students taking an anamnesis, students performing physical exam and students performing medical procedures (under the supervision of a physician-instructor).

We compared level of agreement across several demographic and professional variables, including: gender (male vs. female), age (≤ 45 years vs. ≥46 years), involvement in teaching (Yes vs. No), religious affiliation (secular vs. religious or traditional Jew), professional role (family medicine resident vs. specialist), and years of experience (≤ 10 years vs. 11+). Categorical levels of agreement were compared using Chi-square tests, while total agreement scores were compared using t-tests.

We tested questionnaire reliability using Cronbach’s Alpha scores. Correlations between total agreement sub-scores was evaluated using Spearman’s rank correlation. *P* < 0.05 was considered statistically significant in all tests.

The work was carried out in accordance with the Declaration of Helsinki, including, but not limited to the anonymity of participants being guaranteed and the informed consent of participants being obtained. The study was approved by the Human Subjects Research Committee of our university - Faculty of Health Sciences, May 16, 2024 (Approval #08-2024).

That committee exempted the study from signing inform consent forms.

All the participants agreed to participate in the study before they filled the electronic questionnaire.

## Results

### Study population characteristics

A total of 149 physicians participated in the study (Table 1). The mean age of the participants was 47.2 (SD 11.6) years with 97 (65.1%) identifying as female. In terms of religious affiliation 98 (65.8%) identified as secular Jews. Professionally, 33 (22.3%) were family medicine residents, while 86 (58.1%) were family medicine specialists. Most of the participants (*n* = 127, 86.4%) had studied medicine in Israel. Regarding years of experience 59 (39.6%) had more than 20 years of experience. The majority (*n* = 118, 79.2%) were involved in teaching students and trainees. Notably, Arab physicians were underrepresented in our sample, likely due to distribution via Hebrew-language CME platforms and social networks.

**Table 1:** Characteristics of the study population (*N* = 149)

	*N*	%
**Age**		
≤ 45 years	77	52.0%
≥ 46 years	71	48.0%
Mean ± Std	47.2 ± 11.6	
Median	45	
Range	30–77	
	148	(mis = 1)
**Gender**		
Male	52	34.9%
Female	97	65.1%
	149	
**Religious affiliation**		
Secular Jew	98	65.8%
Religious or traditional Jew	42	28.2%
Muslim	5	3.4%
Christian	1	0.7%
Other	3	2.0%
	149	
**Professional role**		
Family medicine resident	33	22.3%
Family medicine specialist	86	58.1%
Other	29	19.6%
	148	(mis = 1)
**Country of Medical Studies**		
Israel	127	86.4%
Other	20	13.6%
	147	(mis = 2)
**Years of experience in the field**		
Up to 5	41	27.5%
6–10	27	18.1%
11–20	22	14.8%
Above 20	59	39.6%
	149	
**Involvement in teaching**		
Yes	118	79.2%
No	31	20.8%
	149	

Std-Standard deviation

Both study groups completed the questionnaire digitally—one recruited online via email, the other face-to-face but given the same digital link. As all responses were submitted online, we lack precise data and cannot assess differences between the study groups.

### Questionnaire reliability

Supplementary Material 2 presents the results of questionnaire reliability exam through Cronbach’s Alpha scores. The Cronbach’s Alpha score of the total questionnaire was high 0.863, the sub-scales Cronbach’s Alpha scores were also robust (0.623–0.830).

### Physicians’ willingness to involve medical students in their care during training

#### Total population

Table 2 illustrate physicians’ willingness to involve medical students in their care during training. Most physicians were comfortable with students passively observing history-taking (*n* = 138, 92.6%) and non-intimate physical exams (*n* = 141, 95.3%). However, this comfort significantly declined for intimate exams, with only 44 respondents (29.5%) comfortable regardless of the student’s gender, and 72 (48.3%) comfortable if the student was of the same gender. When it came to answering medical questions, 138 of physicians (92.6%) were open to answering general questions, and 133 (89.3%) were willing to address specific topics like substance use. However, the willingness dropped to 105 (71.4%) for questions regarding sexual habits. In terms of physical exams, 106 (71.1%) of physicians agreed to be examined for educational purposes though fewer (*n* = 28, 19.0%) agreed to intimate exams. While 128 (85.9%) were comfortable with students performing non-invasive procedures under supervision, only 25 (16.8%) agreed to invasive procedures involving intimate areas. Preference for a same-gender student led to only a minimal increase in acceptance (*n* = 26, 17.6%), a difference that is not statistically or practically significant. There was a statistically significant positive correlation between all attitude scores as shown in Supplementary material 3. The strongest correlation was found between the passive participation attitudes score and the performing a medical procedure score, *r* = 0.504, *p* < 0.001. Although the correlations between attitude scores were statistically significant, the proportion of explained variance was moderate (e.g., *r* = 0.504; r² = 0.25), suggesting that the different attitude domains are related but conceptually distinct.

**Table 2:** Physicians’ willingness to involve medical students in their care during training

	Level of agreement							
	Disagree		Neutral		Agree		Not relevant	
	*N*	%	*N*	%	*N*	%	*N*	%
**Physicians’ willingness for student observation during medical sessions**								
History-taking	2	1.3%	9	6.0%	138	92.6%	0	0.0%
Non-intimate physical exams	2	1.4%	5	3.4%	141	95.3%	0	0.0%
Intimate physical exams-regardless of the student’s gender	77	51.7%	28	18.8%	44	29.5%	0	0.0%
Intimate physical exams-student of same gender	51	34.2%	26	17.4%	72	48.3%	0	0.0%
**Physicians’ willingness to answer student questions during anamnesis**								
Answers the student’s questions	4	2.7%	7	4.7%	138	92.6%	0	0.0%
Answers the student’s questions-student of same gender	100	68.5%	13	8.9%	33	22.6%	0	0.0%
Answers questions regarding habits such as substance use: alcohol, drugs	8	5.4%	8	5.4%	133	89.3%	0	0.0%
Answers questions about sexual habits and sexual preference-regardless of the student’s gender	26	17.7%	16	10.9%	105	71.4%	0	0.0%
Answers questions about sexual habits and sexual preference-student of same gender	36	24.2%	17	11.4%	96	64.4%	0	0.0%
**Physicians’ willingness for student physical examination**								
Any physical examination	17	11.4%	24	16.1%	106	71.1%	2	1.3%
Any physical examination -student of same gender	46	31.1%	24	16.2%	67	45.3%	11	7.4%
Breast, genital, or rectal examination-regardless of the student’s gender	95	64.6%	23	15.6%	28	19.0%	1	0.0%
Breast, genital, or rectal examination-student of same gender	81	55.5%	21	14.4%	39	26.7%	5	3.4%
**Physicians’ willingness for student procedures under supervision**								
Any procedure performed under the guidance of a physician-instructor according to the student’s learning needs	49	32.9%	44	29.5%	56	37.6%	0	0.0%
A procedure that does not involve risk (insertion of a transfusion, taking blood)	10	6.7%	10	6.7%	128	85.9%	1	0.7%
A procedure that does not require the exposure of intimate organs (suturing an incision, draining abscesses)	49	32.9%	26	17.4%	74	49.7%	0	0.0%
A procedure that involves exposing intimate organs -insertion of a catheter into the bladder -regardless of the student’s gender	97	65.1%	27	18.1%	25	16.8%	0	0.0%
A procedure that involves exposing intimate organs -insertion of a catheter into the bladder-student of same gender	97	65.5%	21	14.2%	26	17.6%	4	2.7%

### Physicians’ willingness to involve medical students in their care during training-Subgroups

A gender-based comparison of physicians’ willingness to be examined by medical students during their training revealed a few notable differences. Female physicians were less willing than their male counterparts to undergo physical examinations by students during clinic or hospital visits (*n* = 61, 63.5% of females vs. *n* = 45, 88.2% of males; *p* = 0.006). A substantial percentage of female physicians reported discomfort with consenting to examinations involving intimate areas when the student was female (28.1%), and an even greater percentage reported discomfort when the student was male (48%). (*n* = 69, 71.9% of females vs. *n* = 26, 52.0% of males; *p* = 0.014).

Physicians involved in teaching were significantly less likely to agree to having medical students present during intimate physical examinations compared to those not involved in teaching (*n* = 16, 51.6% vs. *n* = 35, 29.7%, *p* = 0.018).

Younger physicians (≤ 45 years) compared to older physicians (≥ 46 years) were less willing to allow students to observe (*n* = 47, 61.0% vs. *n* = 30, 42.3%, *p* = 0.033) or perform intimate exams (*n* = 56, 73.7% vs. *n* = 38, 55.1%, *p* = 0.016). Younger physicians were also more likely to answer questions about sexual habits if the student was of the same gender (*n* = 56, 72.7% vs. *n* = 40, 56.3%, *p* = 0.051).

No statistically significant differences were observed based on the physicians’ level of religious observance, professional roles, or years of experience.

In addition, comparisons of the total attitude score and the specific sub-scores across different socio-demographic characteristics showed no statistically significant differences (Supplementary material 4).

## Discussion

### Key findings

Our findings reveal distinct patterns in physicians’ attitudes toward medical student involvement in their care.

The finding that teaching physicians were less likely to consent to intimate examinations (29.7% vs. 51.6%, *p* = 0.018) likely reflects their heightened awareness of the complexities inherent in teacher-student relationships. These physicians may anticipate future professional encounters with the student, making the situation more personally and professionally sensitive than for non-teaching physicians or typical patients. This underscores the importance of establishing clear policies and ethical guidelines regarding boundaries in clinical teaching.

Moreover, physicians’ reluctance to undergo intimate examinations or answer personal questions from students raises broader concerns about informed consent in clinical rotations. Many patients do not clearly distinguish between students, interns, and senior physicians and may be unaware of their right to decline student involvement. Studies on sensitive examinations (e.g., pelvic exams under anesthesia) show that many students perform these procedures without explicit patient consent or are unsure whether consent was obtained [14]. The literature highlights the need for more robust and standardized consent processes to respect patient autonomy and align with ethical standards [15]. Teaching physicians expressed the most discomfort, likely due to role duality and the potential for future encounters with students.

#### Informed consent

in clinical teaching settings is a central ethical concern. The American Medical Association’s Code of Medical Ethics emphasizes that patients must be clearly informed about the presence and role of medical students and must provide explicit consent [2]. When healthcare professionals become patients, the tension between their educational commitment and their personal comfort underscores the need for robust, respectful consent practices.

### Policy implications

Our findings highlight important policy challenges at the intersection of medical education, professional boundaries, and informed consent—especially when the patient is a physician, and particularly a physician-teacher. The results suggest that this unique patient group experiences discomfort with medical student involvement in intimate care situations, raising ethical, educational, and institutional concerns that warrant direct policy attention.

1. **Patient Assignment Protocols**

Physician-patients represent a small fraction of the overall patient population. However, their dual role as both providers and educators creates an inherent conflict when they are asked to participate in medical student training. One policy approach would be to automatically exclude physician-teachers from being assigned to medical students for intimate examinations. This may reduce discomfort and avoid blurred professional boundaries. The downside is that such a policy could be viewed as a double standard—where faculty who promote patient participation in student education are exempting themselves from the same expectation.

A more balanced policy might include offering physician-patients the *option* to decline student involvement proactively, with institutional support normalizing and legitimizing this choice. This avoids mandatory exclusion while ensuring autonomy and minimizing coercion. Clear messaging that opting out is acceptable may reduce social pressure on faculty-patients to “set an example” at the expense of personal comfort.

2. **Informed consent procedures**

Our data suggest that even physicians may not always feel fully empowered to refuse student involvement, particularly in sensitive examinations. This reflects a broader concern in clinical education settings, where patients often feel unable or uncomfortable declining student participation. Medical schools and teaching hospitals should implement standardized and transparent informed consent procedures for all patients—including physician-patients—that explicitly address the presence and role of medical students in care.

These procedures should go beyond generic consent at admission and include real-time, verbal, and specific consent before student participation in physical or intimate examinations. Consent processes should also differentiate between student involvement in observation vs. hands-on examination, especially in sensitive contexts. The process should be led by someone not directly involved in student evaluation to reduce perceived pressure. While developing a universal consent form was beyond the scope of this study, our findings highlight the need for a dedicated effort in this direction. We recommend the establishment of a multidisciplinary working group—including physicians, medical students, and clinical education leaders—to examine the specific concerns raised by physician-patients. This group could synthesize insights from our study and related future research to develop appropriate policies and practical tools, such as tailored consent forms, to support ethical and effective clinical teaching.

3. **Professional Boundary Guidelines**

Physician-teachers may face unique discomfort when examined by their current or future students. Institutions should develop clear professional boundary guidelines that address this dynamic. One possible approach is to explicitly discourage teacher-student examination encounters unless specifically initiated and consented to by the physician-patient. Policies should clarify that declining student involvement will not affect the student’s standing, nor the teacher’s relationship with the institution.

At the same time, faculty development programs should prepare educators to model ethical boundary-setting, including the right to refuse. If carefully framed, such behavior can itself become a valuable teaching moment for students on professionalism and consent.

### Study limitations

The use of convenience sampling introduces potential bias and limits generalizability, as reflected in the overrepresentation of Israeli medical school graduates (86% in our sample versus approximately 40% nationally). Arab physicians were also underrepresented, likely due to language barriers, cultural sensitivities around intimate topics, and recruitment through academic networks more accessible to Israeli-trained physicians. These network effects may have excluded clinicians less engaged in academic or professional forums. Additionally, the small number of Muslim respondents limits our ability to draw conclusions about this culturally distinct subgroup.

The questionnaire used was not validated against existing instruments, and the scenarios presented were hypothetical, which may not fully capture real-world behavior. Despite these limitations, convenience sampling can yield valuable insights in policy-oriented research. The consistent discomfort expressed by physician-patients regarding certain student interactions—particularly intimate examinations—highlights the need for institutional policy responses, even in the absence of a fully representative sample.

While our data support actionable policy steps, qualitative research could deepen understanding of physician-patients’ decision-making and support effective implementation.

## Conclusions

Our findings underscore the need for institutional policies that safeguard physician-patient autonomy, define professional boundaries in teaching settings, and ensure robust informed consent. The proposed recommendations aim to guide ethical and practical improvements in clinical education.

## Supplementary Information

Below is the link to the electronic supplementary material.

Supplementary Material 1: Study Questionnaire

Supplementary Material 2: Cronbach's Alpha reliability scores

Supplementary Material 3: Correlations between attitude sub-scores

Supplementary Material 4: Detailed demographic comparisons

## Data Availability

The ethics committee does not approve sharing the data.
