# Supplementary material for: Policy implications of physicians’ attitudes towards being examined by medical students
Source: Isr J Health Policy Res. 2025 Aug 13;14:50. doi: 10.1186/s13584-025-00711-6 (PMC12344858; doi:10.1186/s13584-025-00711-6)
Supplement: Supplementary file 1 — Supplementary Material 1: Study Questionnaire [file 13584_2025_711_MOESM1_ESM.docx]

**Supplementary material 1:** Study Questionnaire

Dear Participants,

You are invited to participate in a study examining doctors' willingness to receive treatment from medical students during their medical training. Thank you for your cooperation.

| **Section** | **Question** | **Scale (1-5)** |
| --- | --- | --- |
| **Observing Medical Sessions** | If you are seeking treatment at a clinic or hospital (as a patient), to what extent do you agree that a medical student may passively participate (as an observer) in the medical session as part of their learning process? | (1 = Strongly Disagree, 5 = Strongly Agree) |
|  | The student observes the anamnesis being taken. | 1 2 3 4 5 |
|  | The student observes a physical examination that does not include an intimate examination. | 1 2 3 4 5 |
|  | The student observes an intimate physical examination (breasts, genitals) performed by the doctor, regardless of the student's gender. | 1 2 3 4 5 |
|  | The student observes an intimate physical examination performed by the doctor when the student is of the same gender as you. | 1 2 3 4 5 |
| **Taking a Medical History** | If you are seeking treatment at a clinic or hospital (as a patient), to what extent do you agree that a medical student may take your anamnesis as part of their learning process? | (1 = Strongly Disagree, 5 = Strongly Agree) |
|  | Answering the student's questions. | 1 2 3 4 5 |
|  | Answering the student's questions when they are of the same gender as you. | 1 2 3 4 5 |
|  | Answering questions regarding habits such as substance use (alcohol, drugs). | 1 2 3 4 5 |
|  | Answering questions about sexual habits and sexual preference, regardless of the student's gender. | 1 2 3 4 5 |
|  | Answering questions about sexual habits and sexual preference when the student is of the same gender as you. | 1 2 3 4 5 |
| **Physical Examination** | If you are seeking treatment at a clinic or hospital (as a patient), to what extent do you agree to be physically examined by a medical student as part of their learning process? | (1 = Strongly Disagree, 5 = Strongly Agree) |
|  | Not relevant. | ☑️ |
|  | Any physical examination according to the student's learning needs. | 1 2 3 4 5 |
|  | Any physical examination if the student is of the same gender as you. | 1 2 3 4 5 |
|  | Breast, genital, or rectal examination, regardless of the student's gender. | 1 2 3 4 5 |
|  | Breast, genital, or rectal examination if the student is of the same gender as you. | 1 2 3 4 5 |
| **Performing Medical Procedures** | If you are seeking treatment at a clinic or hospital (as a patient), to what extent do you agree that a medical student may perform a medical procedure on you under the supervision of a physician-instructor? | (1 = Strongly Disagree, 5 = Strongly Agree) |
|  | Not relevant. | ☑️ |
|  | Any procedure performed under the guidance of a physician-instructor, according to the student's learning needs. | 1 2 3 4 5 |
|  | A procedure that does not involve risk (e.g., insertion of a transfusion, taking blood). | 1 2 3 4 5 |
|  | A procedure that does not require the exposure of intimate organs (e.g., suturing an incision, draining abscesses). | 1 2 3 4 5 |
|  | A procedure that involves exposing intimate organs (e.g., insertion of a bladder catheter), regardless of the student's gender. | 1 2 3 4 5 |
|  | A procedure that involves exposing intimate organs (e.g., insertion of a bladder catheter) if the student is of the same gender as you. | 1 2 3 4 5 |

**Socio-Demographic Questions**

| **Question** | **Options** |
| --- | --- |
| What is your age? | _____________ |
| What is your gender? | ☐ Male ☐ Female ☐ Other / Prefer not to answer |
| Country of birth? | ☐ Israel ☐ Other: ___________ |
| How do you define yourself religiously? | ☐ Secular Jew ☐ Religious or Traditional Jew ☐ Muslim ☐ Christian ☐ Other |
| What is your profession? | ☐ Family Medicine Resident ☐ Family Medicine Specialist ☐ Other |
| Country of Medical Studies? | ☐ Israel ☐ Other: ___________ |
| Years of experience in the field? | ☐ Up to 5 ☐ 6–10 ☐ 11–20 ☐ More than 20 |
| Do you teach students and residents? | ☐ Yes ☐ No |

**Thank you for your participation!**
