# Supplementary material for: Policy implications of physicians’ attitudes towards being examined by medical students
Source: Isr J Health Policy Res. 2025 Aug 13;14:50. doi: 10.1186/s13584-025-00711-6 (PMC12344858; doi:10.1186/s13584-025-00711-6)
Supplement: Supplementary file 2 — Supplementary Material 2: Cronbach's Alpha reliability scores [file 13584_2025_711_MOESM2_ESM.docx]

**Supplementary material 2: Cronbach's Alpha reliability scores**

| **Sub-scale** | **N of Items** | **Cronbach's Alpha** | **Item** | **Cronbach's Alpha if Item Deleted** |
| --- | --- | --- | --- | --- |
| Passive participation of the student (as an observer) in the medical session | 4 | 0.724 | The student watches the anamnesis being taken | 0.859 |
|  |  |  | The student watches the physical examination that does not include an intimate examination | 0.862 |
|  |  |  | The student watches an intimate physical examination: breasts, genitals performed by the doctor, regardless of the student's gender | 0.851 |
|  |  |  | The student watches an intimate physical examination performed by the doctor when the student is of the same gender as me | 0.851 |
| The student takes anamnesis | 5 | 0.623 | Answers the student's questions | 0.858 |
|  |  |  | Answers the student's questions whether they are of the same gender as me | 0.875 |
|  |  |  | Answers questions regarding habits such as substance use: alcohol, drugs. | 0.857 |
|  |  |  | Answers questions about sexual habits and sexual preference regardless of the student's gender | 0.852 |
|  |  |  | Answers questions about sexual habits and sexual preference when the student is of the same gender as me. | 0.859 |
| The student performs a physical examination | 4 | 0.657 | Any physical examination according to the student's learning needs | 0.856 |
|  |  |  | Any physical examination if the student is of the same gender as me | 0.861 |
|  |  |  | Breast, genital, or rectal examination regardless of the student's gender | 0.853 |
|  |  |  | Genitalia, breast, or rectal examination - if the student is of the same gender | 0.854 |
| The student performs medical procedure (under the supervision of a physician-instructor) | 5 | 0.830 | Any procedure performed under the guidance of a physician-instructor according to the student's learning needs | 0.851 |
|  |  |  | A procedure that does not involve risk, such as: insertion of a transfusion, taking blood | 0.858 |
|  |  |  | A procedure that does not require the exposure of intimate organs such as: suturing an incision, draining abscesses | 0.855 |
|  |  |  | A procedure that involves exposing intimate organs such as: insertion of a catheter into the bladder. Regardless of the student's gender | 0.849 |
|  |  |  | A procedure that involves exposing intimate organs such as: insertion of a catheter into the bladder if the student is of the same gender as me | 0.851 |
| **Total** | **18** | **0.863** |  |  |
