# Supplementary material for: Policy implications of physicians’ attitudes towards being examined by medical students
Source: Isr J Health Policy Res. 2025 Aug 13;14:50. doi: 10.1186/s13584-025-00711-6 (PMC12344858; doi:10.1186/s13584-025-00711-6)
Supplement: Supplementary file 3 — Supplementary Material 3: Correlations between attitude sub-scores [file 13584_2025_711_MOESM3_ESM.docx]

Supplementary material 3**:** **Correlations between attitude sub-scores**

|  | | **Passive participation attitudes score** | **Anamnesis taking attitudes score** | **Performing a physical examination score** | **Performing a medical procedure score** |
| --- | --- | --- | --- | --- | --- |
| **Passive participation attitudes score** | r | 1 |  |  |  |
|  | P-value |  |  |  |  |
|  | N | 149 |  |  |  |
| **Anamnesis taking attitudes score** | r | .373 | 1 |  |  |
|  | P-value | <0.001 |  |  |  |
|  | N | 149 | 149 |  |  |
| **Performing a physical examination score** | r | .490 | .429 | 1 |  |
|  | P-value | <0.001 | <0.001 |  |  |
|  | N | 149 | 149 | 149 |  |
| **Performing a medical procedure score** | r | .504 | .352 | .434 | 1 |
|  | P-value | <0.001 | <0.001 | <0.001 |  |
|  | N | 149 | 149 | 149 | 149 |
