# Supplementary material for: Policy implications of physicians’ attitudes towards being examined by medical students
Source: Isr J Health Policy Res. 2025 Aug 13;14:50. doi: 10.1186/s13584-025-00711-6 (PMC12344858; doi:10.1186/s13584-025-00711-6)
Supplement: Supplementary file 4 — Supplementary Material 4: Detailed demographic comparisons [file 13584_2025_711_MOESM4_ESM.docx]

Supplementary material 4: Detailed demographic comparisons

| **sociodemographic**  **characteristics** | N | **Total attitude**  **score** | | | **Passive participation attitudes score** | | | **Anamnesis taking attitudes score** | | | **Performing a physical examination score** | | | **Performing a medical procedure score** | | |
| --- | --- | --- | --- | --- | --- | --- | --- | --- | --- | --- | --- | --- | --- | --- | --- | --- |
|  |  | **Mean** | **Std** | **pvalue** | **Mean** | **Std** | **pvalue** | **Mean** | **Std** | **pvalue** | **Mean** | **Std** | **pvalue** | **Mean** | **Std** | **pvalue** |
| **Age** |  |  |  |  |  |  |  |  |  |  |  |  |  |  |  |  |
| ≤45 years | 77 | 59.7 | 12.9 | 0.535 | 15.0 | 3.2 | 0.369 | 19.1 | 4.2 | 0.478 | 11.3 | 4.2 | 0.720 | 14.2 | 5.1 | 0.204 |
| ≥46 years | 71 | 60.9 | 11.8 |  | 15.5 | 3.5 |  | 18.6 | 4.1 |  | 11.5 | 3.8 |  | 15.3 | 4.5 |  |
| **Gender** |  |  |  |  |  |  |  |  |  |  |  |  |  |  |  |  |
| Male | 52 | 62.0 | 11.7 | 0.211 | 15.5 | 3.4 | 0.468 | 19.1 | 3.9 | 0.688 | 12.2 | 4.0 | 0.074 | 15.2 | 4.8 | 0.373 |
| Female | 97 | 59.3 | 12.6 |  | 15.1 | 3.3 |  | 18.8 | 4.2 |  | 11.0 | 3.9 |  | 14.5 | 4.9 |  |
| **Religious affiliation** |  |  |  |  |  |  |  |  |  |  |  |  |  |  |  |  |
| Secular Jew | 98 | 60.9 | 13.2 | 0.643 | 15.4 | 3.3 | 0.194 | 18.9 | 4.5 | 0.638 | 11.4 | 4.1 | 0.640 | 15.2 | 4.9 | 0.258 |
| Religious or traditional Jew | 42 | 58.8 | 10.8 |  | 14.6 | 3.2 |  | 18.6 | 3.4 |  | 11.6 | 3.7 |  | 14.0 | 4.8 |  |
| Other | 9 | 59.9 | 8.2 |  | 16.6 | 3.1 |  | 20.0 | 2.8 |  | 10.2 | 3.7 |  | 13.1 | 4.2 |  |
| **Professional role** |  |  |  |  |  |  |  |  |  |  |  |  |  |  |  |  |
| Family medicine resident | 33 | 60.8 | 11.4 | 0.403 | 15.3 | 3.5 | 0.261 | 19.4 | 3.3 | 0.387 | 11.5 | 4.1 | 0.338 | 14.5 | 4.9 | 0.958 |
| Family medicine specialist | 86 | 61.0 | 11.7 |  | 15.5 | 3.2 |  | 18.9 | 3.9 |  | 11.8 | 3.8 |  | 14.8 | 4.9 |  |
| Other | 29 | 57.5 | 15.1 |  | 14.3 | 3.3 |  | 18.0 | 5.5 |  | 10.5 | 4.2 |  | 14.7 | 5.0 |  |
| **Country of Medical Studies** |  |  |  |  |  |  |  |  |  |  |  |  |  |  |  |  |
| Israel | 127 | 60.1 | 12.0 | 0.561 | 15.3 | 3.3 | 0.901 | 18.7 | 4.0 | 0.332 | 11.4 | 3.9 | 0.799 | 14.7 | 4.7 | 0.592 |
| Other | 20 | 61.9 | 14.0 |  | 15.2 | 3.3 |  | 19.7 | 4.5 |  | 11.6 | 4.8 |  | 15.4 | 5.9 |  |
|  |  |  |  |  |  |  |  |  |  |  |  |  |  |  |  |  |
| **Years of experience in the field** |  |  |  |  |  |  |  |  |  |  |  |  |  |  |  |  |
| Up to 5 | 41 | 60.4 | 10.9 | 0.986 | 15.3 | 3.0 | 0.903 | 19.0 | 3.5 | 0.777 | 11.3 | 4.1 | 0.986 | 14.8 | 4.4 | 0.780 |
| 6-10 | 27 | 60.6 | 15.9 |  | 15.0 | 3.8 |  | 19.4 | 5.1 |  | 11.6 | 4.6 |  | 14.6 | 5.7 |  |
| 11-20 | 22 | 59.4 | 12.5 |  | 14.9 | 3.2 |  | 19.1 | 3.9 |  | 11.5 | 4.1 |  | 13.9 | 5.3 |  |
| Above 20 | 59 | 60.3 | 11.6 |  | 15.4 | 3.4 |  | 18.5 | 4.2 |  | 11.3 | 3.6 |  | 15.1 | 4.7 |  |
| **Involvement in teaching** |  |  |  |  |  |  |  |  |  |  |  |  |  |  |  |  |
| Yes | 118 | 60.8 | 12.0 | 0.336 | 15.4 | 3.2 | 0.184 | 18.8 | 4.0 | 0.809 | 11.6 | 3.8 | 0.315 | 14.9 | 4.9 | 0.361 |
| No | 31 | 58.4 | 13.3 |  | 14.5 | 3.7 |  | 19.0 | 4.5 |  | 10.8 | 4.6 |  | 14.0 | 4.6 |  |

***** Bonferroni correction was applied for multiple comparisons (35 tests). Adjusted significance threshold: p < 0.0014. No comparisons remained significant after correction
